# Supplementary material for: Sequence and expression levels of circular RNAs in progenitor cell types during mouse corticogenesis
Source: Life Sci Alliance. 2019 Mar 29;2(2):e201900354. doi: 10.26508/lsa.201900354 (PMC6441494; doi:10.26508/lsa.201900354)
Supplement: Supplementary file 2 [file LSA-2019-00354_TableS1.docx]

**Table S1**. **Primer List**. (**A**) List of divergent primers for all the tested circRNAs in **Figure 1C**. (**B**) Primer used for cloning and sequencing in Figure 1B. (**C**) Primers for linear transcript used in RNase R efficacy test, **Figure S1A**. (**D**) List of divergent primers for intron validation, **Figure S1C**.

(**A**)

| CiCo_mm9_circ_ |  | Sequence | Expected size |
| --- | --- | --- | --- |
| 001074 | FWD | GATGTGAGCCTGACTGAACG | 147 |
|  | REV | CTGCTGCTGGTTCTTGCTC |  |
| 001268 | FWD | CAGCTTGGCATGTGGAAAT | 146 |
|  | REV | CAGAGAAGAGCCCCTGGAC |  |
| 001423 | FWD | GCCTTGTTCTTCTGGTGAGC | 154 |
|  | REV | CTCCTGAACAGAAGGCTTGAA |  |
| 001527 | FWD | CCCTGAAAATGCAGAGAGAA | 142 |
|  | REV | TCTTGTTGGGCACAAAGATG |  |
| 001902 | FWD | CAAGTGTGACAGGCGCATAA | 143 |
|  | REV | CACCTGGTGGTTGATCTTGA |  |
| 002029 | FWD | CCAACTACGCAGACCCCTAC | 148 |
|  | REV | TCTGCTGAACTGGGGTCTTT |  |
| 000203 | FWD | CGCGAGGGACTGACAGAGT | 146 |
|  | REV | AGGGTCCGTGCGAAGAAG |  |
| 002131 | FWD | GAACCCCAAGGCTCTGCT | 149 |
|  | REV | GAACAGTTGGTGACCACGAG |  |
| 002132 | FWD | CCTGCTGGACCTCTCAGG | 149 |
|  | REV | GGATCTCAGCCAGAGACATGA |  |
| 002204 | FWD | GGACAGTGAGGAGCTCAGG | 153 |
|  | REV | AGGCTCCGAAGAAAGTGCT |  |
| 002231 | FWD | AAGAGCAGGGCATCATCTCT | 149 |
|  | REV | TCTTTGGCAAGCTGTGGTC |  |
| 002232 | FWD | GGGATGCATCTCTTGATAACTG | 147 |
|  | REV | TCCTCCAGATCTCTGTGGAAT |  |
| 002259 | FWD | ATCGGAGTTTTGGACCAATC | 145 |
|  | REV | TTGCTCGACTCATAGCTGGA |  |
| 002390 | FWD | CAACATCTCCTCGGATGTCA | 145 |
|  | REV | AGGCCACTTTTGAGTTCTTGTC |  |
| 003161 | FWD | CTCACGGGGCTCTGTCAAG | 149 |
|  | REV | GGAAGCTCCCATCAGGAAAT |  |
| 003578 | FWD | AAACGCTGACATCGAGCTG | 156 |
|  | REV | GCGATCGGTTCTTAGCACTC |  |
| 003934 | FWD | CAGCAACCACCAGCTCCTAT | 145 |
|  | REV | GGCCTGTGATATCATTCTGCT |  |
| 003968 | FWD | GGCCCAAACCCTTAAAATTAC | 145 |
|  | REV | TTTGCATCTTGCTCCCTCAT |  |
| 004187 | FWD | CCTCTCCACCACTGTCAGC | 150 |
|  | REV | CATGTCCAGTTCCTCTGAAGAT |  |
| 004202 | FWD | GGAAGATGAGGACGAAGATGA | 143 |
|  | REV | TGTCACCTGAATTTCGTCTTTC |  |
| CiCo_mm9_circ_ |  | Primer sequence | Expected size |
| 004254 | FWD | TTACTGTGAAGTTTGCCAACAA | 144 |
|  | REV | TGAGGCTCCATGGTGCTAAT |  |
| 004860 | FWD | AACACCATCACTCGGCTAAAG | 147 |
|  | REV | TGTGCCCAGGATGTTACAGA |  |
| 004878 | FWD | ACGTTCCTGCCTGAGCTG | 153 |
|  | REV | CACAGGTGCCTTGGTAAGGT |  |
| 005387 | FWD | TTGGAGAAAATGCTTCCAGA | 148 |
|  | REV | CCAGGATTCAAGCCAGTGTC |  |
| 005420 | FWD | TCCCAGGAAGAATTACAAAGG | 171 |
|  | REV | TCTGAAGACTTCTGGGTCTGC |  |
| 005430 | FWD | GGCTGTCTGACTGGTGGAAC | 147 |
|  | REV | CTGTGGACCCAGTGGTGAC |  |
| 006088 | FWD | GACTGGCCAGGGGACTTC | 143 |
|  | REV | CGGTGGCACCAGAGTGAC |  |
| 006218 | FWD | ACCTCTGGGCAGAACAGC | 141 |
|  | REV | GTTCCAGATCAAATCCCTTGA |  |
| 006222 | FWD | GGGCCATGGTATCTCTGTGT | 147 |
|  | REV | CCAGGAATTAGCCAGGATTTG |  |
| 000669 | FWD | GCCTGTCTACATCAACATCATC | 151 |
|  | REV | GAAGGGAAGGACCAGGTAGC |  |
| 000859 | FWD | ATGAACCAGCTTTCCCTCCT | 145 |
|  | REV | TAGCTGACGAGCCCTCTCTC |  |
| cdr1as | FWD | CTCCAGTGTATCGGCGTTTT | 153 |
|  | REV | TCACGATTGTCTGGAAGACCT |  |
| 000202 | FWD | GCCATTCAGGCTCATCAATA | 156 |
|  | REV | GTCTCGGTCATTCCACGACT |  |
| 000239 | FWD | GCTACCCCAGCTCCAACAT | 147 |
|  | REV | GCTTAAGAGGGCTGTGCTGT |  |
| 005390 | FWD | ACAGCAGACAGCTCCAATCA | 150 |
|  | REV | TTGGAAAGATGGGTGTTGGT |  |
| 006087 | FWD | CCCTGAACGACTGTATGCAC | 149 |
|  | REV | GGCACGGAAATCCAAGCTAT |  |
| 002232 | FWD | TGTGGCCTGTGTATGAAGGA | 151 |
|  | REV | CTCCAGAGGCCAGTAAGTTCC |  |
| 003872 | FWD | TCCTTCTTCAGCAAACACAACA | 149 |
|  | REV | TGAGATTTCGAGCTTGTTTGG |  |
| 000720 | FWD | GCTTCAACTGGAATGGCAAG | 154 |
|  | REV | TCTGGGCCATGTCTGAATAA |  |
| 000721 | FWD | TGTGCCTTCATTGCACATT | 155 |
|  | REV | TCTGGGCCATGTCTGAATAA |  |
| circEzh2 | FWD | TTACACGCTTCCGCCAAC | 131 |
|  | REV | AAGCAGCGGAGGATACAGC |  |

(**B**)

| CiCo_mm9_circ_ |  | Primer sequence for cloning | Expected size |
| --- | --- | --- | --- |
| 000203 | FWD | ACGTCTCGAGAGGACCCTGCTTTCTCTGCTGTGATTC | 37,144 (701) |
|  | REV | ACGTGTCGACACCGTGCGAAGAAGGAAGCGGCCACTG |  |
| 000720 | FWD | ACTGCTCGAGATACTCAGGTTGATGGAGTCAGGGA | 32,985 (961) |
|  | REV | ACGTTCTAGACTTTGGCTGAGCAAGTTTACAGTTT |  |
| 000721 | FWD | ACTGCTCGAGATACTCAGGTTGATGGAGTCAGGGA | 27,052 (754) |
|  | REV | CAGTGTCGACCTTGATAAGCAGAACTTAGCCATTT |  |
| 003872 | FWD | ACTGCTCGAGCGCCATCATATAGGAGATCGTAGCC | 5,307 (611) |
|  | REV | CAGTGTCGACCAGTTTGTATACAGACTCGTGATGG |  |
| 004202 | FWD | ACTGCTCGAGAGGTGACAGAGTTAGTCCTCGATAATT | 4,131 (603) |
|  | REV | CAGTGTCGACACCTGAATTTCGTCTTTCATTAAGTAT |  |
| 004583 | FWD | ACGTCTCGAGAGCCAGGTCGCACTGCCTGCGTCACTA | 199 |
|  | REV | ACGTGTCGACACGAGGGACATGTTCCTTATCCTTTCG |  |
| 006087 | FWD | ACGTCTCGAGAGGCCTCAGTATGGTGGCAAGTACTGT | 6,529 (673) |
|  | REV | ACGTGTCGACACTTCAAAGACCAGCGTCTCATTCGTG |  |
| 006088 | FWD | ACGTCTCGAGAGGACATTTGCAAGTCACTCTGGTGCC | 17,979 (918) |
|  | REV | ACGTGTCGACACTTCAAAGACCAGCGTCTCATTCGTG |  |
| 006222 | FWD | ACGTCTCGAGAGGCAGGCTAACGAAGAATATCAAATC | 33,824 (490) |
|  | REV | ACGTGTCGACACCACTTGTCCATTGTGTGGGTTCTTA |  |
| 002131 | FWD | ACGTCTCGAGAGGCCCGCACCTCGTGGTCACCAACTG | 2,606 (257) |
|  | REV | ACGTGTCGACACCGTGCGGTTTTTTGACTGCAGCTCA |  |
| 004187 | FWD | ACGTCTCGAGAGAAAATCTTCAGAGGAACTGGACATG | 3,711 (331) |
|  | REV | ACGTGTCGACACCTTTCTCTTCCTCGGAATGGGCTCA |  |
| 005387 | FWD | ACGTACGCGTAGGAGGAAGAATATGGAAAAGACAATG | 17,031 (3,784) |
|  | REV | ACGTTCTAGAACCTGAAATTGGAAAGATGGGTGTTGG |  |
| 005390 | FWD | ACGTGAATTCAGAAAATGCTTCCAGACTGCTCACTTT | 8,903 (296) |
|  | REV | ACGTGTCGACACAGGCTACGATATTGACGTATCTGTT |  |
| 005431 | FWD | ACGTCTCGAGAGAACTTTGTGCGTCACCACTGGGTCC | 87,653 (1,409) |
|  | REV | ACGTGTCGACACTCATTGAGCAAAGGCATCGAGGTTC |  |
| 006631 | FWD | ACGTCTCGAGAGCCATGGAAACAAAGAAGTATTCTCG | 6,546 (375) |
|  | REV | ACGTGTCGACACTTGTCATTGACAAAGGAATACATCA |  |

(**C**)

| Transcript ID |  | Primer sequence | Expected size |
| --- | --- | --- | --- |
| circEzh2 | FWD | TTACACGCTTCCGCCAAC | 131 |
|  | REV | AAGCAGCGGAGGATACAGC |  |
| Ezh2^LIN^ | FWD | GCGGGACTAGGGAGTGTTC | 154 |
|  | REV | TGTAAAACAGTTTCGTCTTCCA |  |
| GAPDH | FWD | AGGTCGGTGTGAACGGATT | 147 |
|  | REV | CGTGAGTGGAGTCATACTGGA |  |
| Transcript ID |  | Primer sequence | Expected size |
| ENSMUST00000045942 Emx1 | FWD | CTCACTCTTTCTTCAGCGCC | 170 |
|  | REV | CGAGAAGGCTGTGCGAATC |  |
| ENSMUST00000056403 H1fx | FWD | CGCACAAGAGCAAGAAGGC | 157 |
|  | REV | GGGCGGATAGGGATAGAGAC |  |
| ENSMUST00000038537 Wtip | FWD | GATGATTCTGCAGGCCCTTG | 164 |
|  | REV | CACAGGAGGCACATTTTGGT |  |
| ENSMUST00000052281 A19Rik | FWD | TATGAGCGTCGGACCTCTTC | 163 |
|  | REV | TCGGGTGCTTGAAGATCACT |  |
| ENSMUST00000154977 Ccdc120 | FWD | TAGGGAGCAGGCGAGGAG | 179 |
|  | REV | GCTGGGCAGACTTACAACAC |  |
| ENSMUST00000012161 Scarf2 | FWD | GGGGATGAGTGTGGGATAGC | 150 |
|  | REV | GTCAGGGCCCCAGAACTG |  |

(**D**)

| CiCo_mm9_circ_ |  | Primer sequence for cloning | Expected size |
| --- | --- | --- | --- |
| 002805 | FWD | ACTGGCTAGCAAGGCAAAATTGGTGTATGAAGAAG | 720 (167) |
|  | REV | CAGTCTCGAGTTTTCGATTTTTCTGATGCTGTAAC |  |
| 004996 | FWD | ACTGGCTAGCACGGACTCAGACATTGAACAAGGAG | 496 (186) |
|  | REV | CAGTCTCGAGTCATCGTCCCATTCAAAGCCTCCGC |  |
| 004228 | FWD | ACTGGCTAGCAGGAGCTCTGGTGGCCTGCTGCATA | 940 (547) |
|  | REV | CAGTCTCGAGAGGTGAAGCGGGCCTGAAGGTAGAG |  |
| 004227 | FWD | ACTGGCTAGCCATGTTCACTATGCTGTATCTGGTG | 883 (490) |
|  | REV | CAGTCTCGAGGGCGTGGCCCGAGAAGAAGGACTTC |  |
| 003247 | FWD | ACTGGCTAGCCCAGCTGCAGACTTTCTCAGAGGAG | 791 (126) |
|  | REV | CAGTCTCGAGGGATTGACAGCAGCCCCCGGTGCTC |  |
| 003141 | FWD | ACTGGCTAGCCCTTGGCGAGTGGCAGCCCCTTGAG | 563 (461) |
|  | REV | CAGTCTCGAGGGTTCCACCAGTTGGCTGAGGCACC |  |
